# Effect of practice on learning to maintain balance under dynamic conditions in children: are there sex differences?

**DOI:** 10.1186/s13102-020-00166-z

**Published:** 2020-03-05

**Authors:** Simon Schedler, Dennis Brueckner, Rainer Kiss, Thomas Muehlbauer

**Affiliations:** 1 Division of Movement and Training Sciences/Biomechanics of Sport, University of Duisburg-Essen, Gladbecker Str. 182, 45141 Essen, Germany; 2 Department of Health and Social Affairs, FHM Bielefeld - University of Applied Sciences, Bielefeld, Germany

**Keywords:** Youth, Skill acquisition, Stabilometer, Postural control, Human

## Abstract

**Background:**

In youth, sex-related differences in balance performances have been reported with girls usually outperforming same-aged boys. However, it is not known whether sex also has an influence on learning of a new balance task in primary school-aged children. Therefore, the present study investigated sex-related differences in children learning to maintain balance under dynamic conditions.

**Methods:**

Thirty-two children (16 girls, 16 boys) aged 8.5 ± 0.5 years practiced balancing on a stabilometer (i.e., to keep it as horizontal as possible) for seven trials (90 s each) on two consecutive days. Knowledge of results (KR) (i.e., time in balance) was provided after each trial. On day three learning was assessed using a retention test (i.e., balance task only) and a test of automation (i.e., balance plus concurrent motor interference task). Root-mean-square-error (RMSE) was recorded for all trials and used for further analysis.

**Results:**

During practicing (Day 1, Day 2) RMSE values significantly decreased over the days (*p* = 0.019, *d* = 0.92) and trials (*p* = 0.003, *d* = 0.70) in boys and girls. Further, the main effect of sex showed a tendency toward significance (*p* = 0.082, *d* = 0.67). On day 3, the girls showed significantly smaller RMSE values compared to boys in the retention (*p* = 0.012, *d* = 1.00) and transfer test (*p* = 0.045, *d* = 0.74).

**Conclusions:**

Performance increases during the acquisition phase tended to be larger in girls than in boys. Further, learning (i.e., retention and automation) was significantly larger in girls compared to boys. Therefore, practitioners (e.g., teachers, coaches) should supply boys and grils with balance exercises of various task difficulties and complexities to address their diverse learning progress.

## Background

Balance is important during activities of daily life as well as in sports and poor balance is associated with an increased risk of falling and sustaining injuries [1]. This is particularly important for children as balance performance does not reach the adult-level before late adolescence due to maturation processes of the postural control system [2]. According to Shumway-Cook and Woollacott [3], a distinction is made between static balance where the base of support and the ground remain stationary and only the center of mass moves (e.g., standing on a firm floor) and dynamic balance where the base of support and/or the ground move and the center of mass shifts (e.g., walking). Further, it is differentiated between proactive (i.e., anticipation of a predicted perturbation) and reactive (i.e., reaction to an unpredicted perturbation) balance. All these components are reportedly independent from one another indicating that a person can exhibit sufficient balance performance during static conditions but perform poor in a dynamic or reactive balance task [4]. As most daily activities (e.g., climbing stairs) are rather dynamic in nature, sufficient balance during dynamic conditions is essential for everyday life.

With respect to balance performance in youth, findings on sex-related differences are rather inconclusive. For example, a systematic review with meta-analysis [2] examining age- and sex-related differences in balance performance in youth found inconsistent results. More precisely, girls showed superior static balance (standardized mean difference [SMD] = 0.33), while boys performed better in proactive conditions (SMD = − 0.15) and almost no difference was found concerning measures of dynamic balance (SMD = − 0.02). Yet, the largest difference was found in favor of girls and this comparison was based on the results of 16 different studies whereas far less studies were available for comparisons of dynamic steady-state balance (*n* = 7) and proactive balance (*n* = 6). Thus, it may be speculated that if existent at all sex-related differences in balance performance in youth may be more likely in favor of girls. In studies which found sex-related differences in balance performance in youth, those have been attributed particularly to the development of the postural control system as girls are known to mature earlier than boys [5, 6]. Yet, there might be other influencing factors such as higher agitation in (younger) boys [6], differences in neural maturation as for instance the total cerebellar volume peaks earlier in girls compared to boys [7], or sports participation (e.g., type and/or amount of sport). However, although girls participate in sports involving lots of balance (e.g., gymnastics) more frequently than boys [8], a study investigating sex differences in balance performance in youth gymnasts [9] still found superior balance performance in female compared to male youth gymnasts.

Practicing a new motor skill leads to short-term adaptations resulting in improved performance in this particular motor skill with repeated trials. If sufficient practice is given during this acquisition phase, long-term adaptations indicating learning can be observed during retention and transfer tests [10]. These tests are usually carried out 24 h after the last practice session so that short-term adaptations from the acquisition phase can dissipate and involve the execution of the practiced task under slightly different conditions (e.g., no feedback) [11]. If actual learning has been achieved, performance under the changed conditions should still be better than at the beginning of the acquisition phase. This relation has been documented in various experiments including fine as well as gross motor skills [10]. Several studies in the field of motor learning applied balance tasks [12–15] using a stabilometer [12, 13], wobbleboards [14], or pedalos [15]. Generally, these studies showed that during the acquisition phase performance improved with practice in adults as well as in children and induced learning of the practiced balance task as indicated by still improved performances in retention and transfer tests. For instance, Becker and Smith [15] observed practice-related improvements in primary school-aged children (8–10 years) and young adults (19–26 years) during 20 practicing trials on a double pedalo followed by a 24 h delayed retention test. More specifically, time to complete a seven meter course on the pedalo significantly decreased with practice and was still improved in the retention test.

Research on motor learning in children using balance tasks has predominantely focused on the influence of focus of attention (internal vs. external) and studies on sex-related differences regarding balance in youth have mostly focused on performance. Yet, besides differences in balance performance between girls and boys there might also be sex-related differences in learning of a balance task. Knowledge about such differences is of major importance for practitioners such as teachers or coaches, for example to specifically design training programs in terms of task difficulty and/or task complexity level. Therefore, the present study aimed to investigate the influence of practice on learning to maintain balance under dynamic conditions in primary school-aged children, also including sex-related differences. Due to the previously reported involvement of maturation, we opted for primary school-aged children to exclude adolescent growth as a potentially confounding factor. We hypothesized, that a) practice leads to short-term adaptations resulting in improved performance in the practiced balance task and that b) improvements will be larger in girls compared to boys. Additionally, we hypothesized that practicing would induce learning and that girls would show better performances than boys in delayed retention and automation tests.

## Methods

### Participants

Thirty-two children (16 boys, 16 girls) with a mean age of 8.5 ± 0.5 years from two local primary schools participated in the study. Table 1 shows group means and standard deviations for chronological age, body height, body mass, leg length, and maturity offset according to sex. Maturity offset was calculated in terms of years from peak height velocity (PHV) for each participant by using the formula provided by Moore et al. [16].

**Table 1:** Characteristics of the children included in the study and *p*-values for comparisons between girls and boys

	Girls (*n* = 16)	Boys (*n* = 16)	*p*-value
Age (years)	8.5 ± 0.5	8.6 ± 0.5	.733
Body height (cm)	136.3 ± 6.3	139.2 ± 6.6	.208
Body mass (kg)	32.0 ± 6.3	33.0 ± 5.8	.650
Leg length (cm)	72.8 ± 4.6	74.8 ± 3.7	.193
Maturity offset^a^ (years from PHV)	−2.81 ± 0.46	−3.69 ± 0.41	.000

Values are presented as means ± standard deviations. ^a^Maturity offset was calculated by using the formula provided by Moore et al. [16]. *PHV* peak height velocity

As verified by participant’s parents report, none of the children had any neurological, orthopedic, or musculoskeletal disorder that could have affected their ability to follow the instructions and/or execute the applied tasks. Furthermore, we assured that none of the children had prior experience with the balance task and/or regularly performed balance training (e.g., tightrope walking) as this could have affected training improvements. All measurements were performed in a separate room within the school building, with only one participant being present at a time.

### Experimental procedure

#### Acquisition (day 1 and 2)

**Fig. 1:**
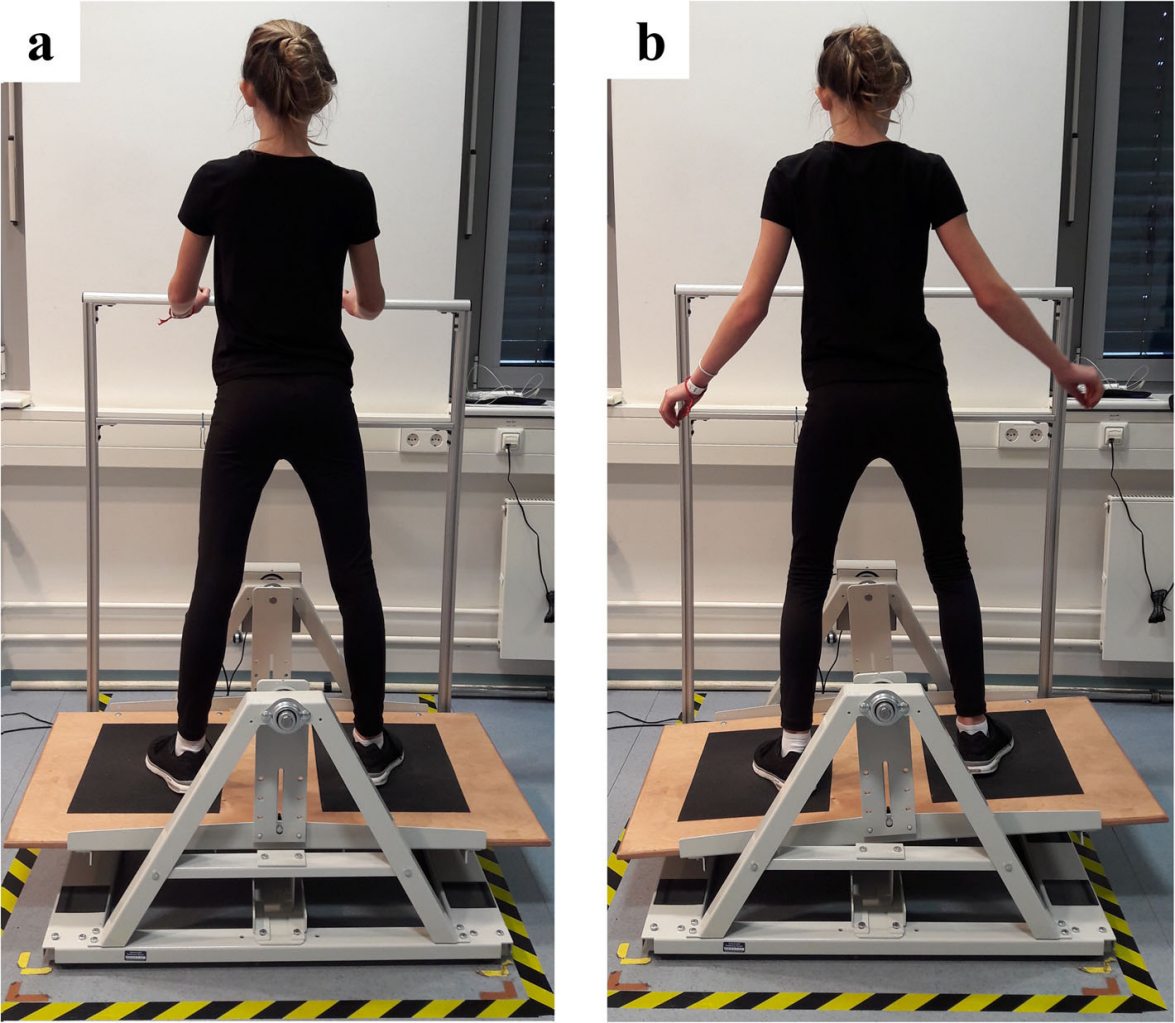
Illustration of a child standing (**a**) and balancing (**b**) on the stability platform (stabilometer). Note. The individual that is shown and its legal guardians have given written informed consent to publish these case details

After anthropometrics were measured, participants were instructed to balance on a stability platform (Lafayette Instrument, Model 16,030, Lafayette, LA, USA). The platform (stabilometer) consisted of a swinging wooden platform (65 × 107 cm) which allowed a maximum deviation of 15 degrees to either side of the horizontal plane of the platform (Fig. 1). To prevent participants from falling in case they lost balance a safety rail mounted to the stabilometer was used.

Participants trained on the stabilometer during two consecutive days. Each session consisted of seven trials lasting 90 s separated by 90 s rest periods. All participants were instructed to balance on the stabilometer in order to keep the platform horizontal (± 3 degrees) while gazing at a fixed target approximately one meter opposite to the platform. Trials started from a horizontal position with participants holding on to the safety rail (Fig. 1a). After each trial, participants received knowledge of results (KR) (i.e., time in balance). Participants had to stepp off of the platform after each trial and were asked to step back on it approximately 20 s before the start of the next trial.

#### Testing (day 3)

Because performance changes during the acquisition phase (days 1 and 2) may only reflect temporary changes, a delayed retention and automation test was carried out to assess learning of the balance task. Procedures during retention test were identical to the acquisition phase, yet participants did not receive KR anymore. Thereafter, an automation test took place, which involved the execution of an additional motor interference task. Participants were asked to hold two connected metal rings in a way that they did not touch each other while balancing on the platform. For the retention and automation test, three trials à 90 s with 90 s rest between trials were performed and no KR was provided.

### Data collecting

A timer sampling platform data at a rate of 25 Hz was used to measure time in balance. A platform angle within ±3 degrees of the horizontal position was defined as ´in balance´. Furthermore, PsymLab Software (Lafayette, LA, USA) was used to export platform position data and calculate the root-mean-square-error (RMSE) of the stability platform angle in degrees which was used for further analyses.

### Statistical analyses

An a priori power analysis using G * Power [17] with the following input parameters was performed to obtain a medium-sized interaction effect: effect size (*d* = 0.50), type I error (*α* = 0.05), type II error (*1-β* = 0.95), number of groups (*n* = 2), number of measurements (*n* = 7), correlation between measurements (*r* = 0.50). Additionally, a dropout rate of 20% was considered. Our analysis revealed a total sample size of 31–32 participants. Descriptive statistics were presented as group means ± standard deviations (SD). Normal distribution was examined using the Shapiro-Wilk test (*p* > 0.05) and homogeneity of variances using the Levene’s test (*p* > 0.05). During acquisition on day 1 and day 2, the RMSE values were analysed in a 2 (sex: boys, girls) × 2 (day: day 1 to 2) × 7 (trial: trial 1 to 7) analysis of variance (ANOVA) with repeated measures on days and trials. During testing on day 3, the RMSE values were separately compared between girls and boys for the retention and automation using a one-way ANOVA. All analyses were adjusted for the observed differences in maturity offset between girls and boys. Additionally, Cohen’s *d* was calculated to determine whether a statistical difference was practically meaningful as small (0 ≤ *d* ≤ 0.49), medium (0.50 ≤ *d* ≤ 0.79), and large (*d* ≥ 0.80). All analyses were performed using the Statistical Package for Social Sciences (SPSS) version 24.0 and significance level was set at *p* < 0.05.

## Results

No significant differences regarding age and anthropometrics (i.e., body height, body mass, leg length) were found between boys and girls. However, girls were significantly closer to reaching PHV than boys (Table 1).

### Acquisition (day 1 and 2)

**Fig. 2:**
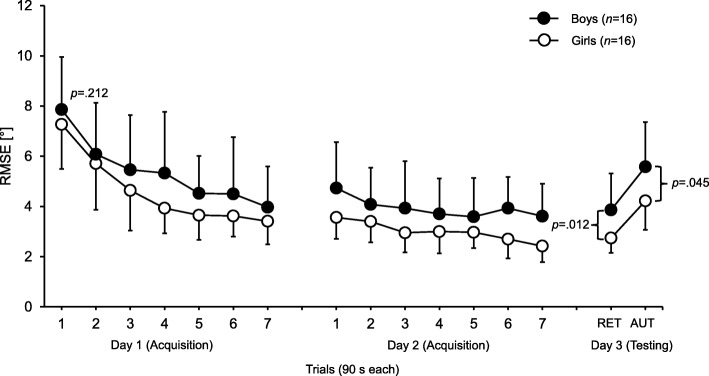
Root mean square error (RMSE) for the boys (filled circles) and girls (unfilled circles) during acquisition (day 1 and day 2) and during testing (day 3). Values represent means and standard deviations. RET = means of three retention test trials (i.e., balance task only); AUT = means of three automation test trials (i.e., balance task plus concurrent motor interference task)

Generally, there were no statistically significant differences (*F*_(1, 30)_ = 1.630, *p* = 0.212) at the start of the experiment (i.e., first trial performance at day 1) between girls and boys. As can be seen from Fig. 2, both the boys and the girls decreased their RMSE values across the 2 days of practice. The adjusted Sex × Day × Trial ANOVA revealed statistically significant main effects of day, *F*_(1, 30)_ = 6.171, *p* = 0.019, *d* = 0.92 and trial, *F*_(6, 180)_ = 3.527, *p* = 0.003, *d* = 0.70. Further, we detetcted a tendency toward significance for the main effect of sex, *F*_(1, 30)_ = 3.247, *p* = 0.082, *d* = 0.67 with girls showing smaller RMSE values during acquisition compared to boys. Yet, we did not find a significant Sex × Day × Trial interaction, *F*_(6, 180)_ = 0.535, *p* = 0.739, *d* = 0.27, indicating that improvements on day 1 and on day 2 were not sex-specific.

### Testing (day 3)

Girls compared to boys showed significantly smaller mean RMSE values in the retention, *F*_(1, 30)_ = 7.248, *p* = 0.012, *d* = 1.00 and automation test, *F*_(1, 30)_ = 3.982, *p* = 0.045, *d* = 0.74 (Fig. 2).

## Discussion

In the present study, we examined the influence of practice on learning to maintain balance under dynamic conditions in primary school-aged children and investigated whether there are differences between girls and boys. Our findings can be summarized as follows: (i) with practice, performance of the trained balance task improved with a tendency toward significantly better performances in girls than in boys; (ii) learning of the new balance task was larger in girls compared to boys, as indicated by their superior performances during retention and automation tests.

The observed increase in performance throughout practicing and subsequent learning of the balance task is in line with previous findings on motor learning in adults [12, 13] as well as in children [18]. However, we found a tendency toward significance but no significant differences in short-term adaptations between boys and girls during the acquisition phase on the first 2 days of practicing. This finding is in contrast to a study of Fujiwara et al. [19]. These authors investigated short-term adaptations to floor oscillations over five consecutive trials in children aged four to 12 years. Results showed that girls were able to adapt to floor oscillations from age five on as indicated by decreased center of pressure (CoP) speed over trials whereas the same development was only seen in boys from age six onwards. Additionally, in 7–8-year-olds CoP speed in girls was significantly smaller at the end of the acquisition phase than in boys. However, our study also included children aged 9 years and Fujiwara et al. [19] did not find sex-related differences in 9–10-year-olds as well. Additionally, although the sex-effect during practicing did not reach significance in our analysis, there was a strong tendency toward it as indicated by the *p*-value of 0.058 and the moderate effect size of 0.72.

Several studies reported associations between neuromuscular activity and improved balance performance [14, 20, 21]. For example, Brueckner et al. [20] used the same stabilometer task in young adult males (26 ± 6 years) and applied surface electromyography (EMG) on the tibialis anterior (TA) and gastrocnemius (GM) muscles. In addition to the performance increase in terms of reduced movement error with practice and subsequent learning, results yielded significantly decreased overall EMG intensity in the TA and GM over the 3 days. Authors concluded that increased movement efficiency during the balance task may account for improved performance in young adults. The same or at least similar mechanisms might explain the present findings in children. However, we abstained from using surface EMG as we examined primary school-aged children in a regular school setting, which delimitated our testing procedures in terms of time and complexity. Moreover, Taubert et al. [22] reported rapid region-specific addaptations of the motor cortex to balance training indicated by significantly increased cortical thickness after a single training session in young adults. Future studies on learning of a balance task in children should therefore at least consider to use surface EMG or functional magnetic resonance imaging (fMRI) to get a better understanding of adaptational processes to motor practice and learning in children.

Both, girls as well as boys, exhibited learning of the new balance task as indicated by their performances during retention and automation tests (Fig. 2). Further, these effects were larger in girls compared to boys. In children, boys reportedly show higher levels of agitation and are usually less attentive than girls especially during balance tasks [23, 24]. This is in line with our observations during the test procedure. For instance, girls stayed quite focused throughout all practice trials trying to balance on the stabilometer as good as possible. Contrary, several boys started asking questions to the experimenter or turned their heads to look around while balancing on the stabilometer. However, especially when practicing a new balance task it is important to concentrate sufficiently. Although, the experimenter did not respond to questions and reminded boys to focus on the mark at the wall as soon as they got distracted these factors may have impeded boys` performance improvements during the acquisition phase to some degree. Nevertheless, they still achieved learning of the task.

Besides these behavioral or psychological explanations, it has been argued that advanced neural maturation may explain superior balance performance of girls. For instance, the total cerebellar volume peaks approximately 2 years earlier in girls than in boys [7]. Similar findings have been reported for gray matter volume [25]. All of these structures are reportedly involved in postural control and balance performance [26]. From a developmental viewpoint, girls in our study were more mature than boys as indicated by the time to PHV and we observed better learning of a new balance task under dynamic conditions in girls compared to boys. Thus, our results corroborate the hypothesis that advanced neural maturation in girls may not only promote balance performance but also facilitate balance learning.

Balance is an important motor skill for children to cope with activities of daily life (e.g., jumping, cycling) as well as in sports performance (e.g., gymnastics, team sports). As balance performance in children is limited due to their still maturing postural control system and poor balance is associated with an increased risk of falling and sustaining an injury, previous research has focused on balance trainability of children. A systematic review with meta-analysis [27] did not find sex to have a significant influence on balance trainability. However, the researchers emphasized the preliminary character of their findings as they observed a lack of high quality studies on this topic. Despite the research on balance trainability in children there has been a void of studies focusing on possible sex differences in children learning a new balance task. In this study, we found that girls show better learning compared to boys. This has important implications for practitioners such as teachers or coaches. On the one hand, boys might need more time to practice a balance task compared to same aged girls. On the other hand, practitioners should have a large repertoire of exercises of various difficulties and/or complexities to keep individuals challenged and facilitate learning progress.

There are a few limitations with this study that have to be addressed. First, we did not apply surface EMG or fMRI and therefore can only speculate on the underlying mechanisms of observed adaptations. Especially, with respect to the differences concerning learning of the balance task between girls and boys, the application of surface EMG or fMRI might have provided deeper insights. Further, we neither assessed participants’ motor nor cognitive development at baseline. As motor as well as cognitive development may have affected participants’ progress in the balancing task, these variables could have been added as covariates in the analysis, thus providing deeper insights into interrelations between motor development, cognitive development, and motor learning. Additionally, the balance task is rather artificial, which limits the transferability of our findings to everyday life situations. Moreover, the presented results only apply to primary school-aged children in the investigated age-group. Future studies are advised to also compare youth of different age-groups as balance performance increases with age. It has been supposed that boys possess an advantage over girls concerning motor skill learning from 9 years onwards, although this finding was limited to manual dexterity [28]. In our study, girls performed significantly better than boys in the delayed retention test. However, this test was carried out 24 h after the last practice and future studies should also investigate sex differences in the performance of the applied balance task following longer retention phases (e.g., 1 week, 1 month). Lastly, possible sex-differences in short-term adaptations (i.e., during practice) need to be clarified as our results indicated a tendency toward a significant sex-effect in favor of girls.

## Conclusions

Practicing a new balance task improved performance and lead to learning of this task in primary school-aged girls and boys. Yet, learning was larger in girls than in boys. Advanced neural maturation in girls as well as higher attention and less agitation might explain these findings. Practitioners such as teachers or coaches should pay attention to these differences when designing training regimes or assessing learning progress and possess a large repertoire of balance exercises with various difficulties and complexities to facilitate learning.

## Data Availability

The data collected and analysed in the present study are not publicly available due to ethical restrictions but are available from the corresponding author upon request.

## Abbreviations

ANOVA: Analysis of variance
CoP: Center of pressure
EMG: Electromyography
fMRI: functional magnetic resonance imaging
KR: Knowledge of results
PHV: Peak height velocity
RMSE: Root-mean-square error
SMD: Standardized mean difference
